# Dresses for Hospital Children

**Published:** 1916-04-08

**Authors:** 


### Dresses for Hospital Children.

The essential features of a good dress for hospital children do not vary and are in no doubt. Plainness and the old-fashioned virtues are the qualities sought. Fabrics warm to wear and slow to burn, easy to clean or mend, long in wearing into holes, reasonably cheap to buy, sensible and homely to look upon, are the ones intrinsically demanded. The practical question is how to lay hold of reliable woollen stuffs which are neither too smart when new nor too shabby when old. The problem has been solved in some quarters by choosing the Ruskin homespuns, made as they have been for thirty-five years in the little country mill at Laxey, Isle of Man, founded by the St. George's Guild, of which John Ruskin was Master.

The goods owe their properties to an absence of sophistication. They are made from the local wool without addition or subtraction, with the aim of producing a simple, unassuming cloth in which the natural merits of the raw material are unimpaired. The wool is carded, the yarns are spun, and the looms are driven by power derived from the waterwheel, and this employment of machinery within its right compass makes the manufacture economical. The avoidance of poor raw materials makes the goods sound. Partly because no addition of short fibre is made, partly also because of the simplicity of the methods employed in finishing the woven cloth, the Ruskin fabrics are of noticeably open texture. They admit fresh air, as the wearer finds in a strong breeze; but also they house warmed air and allow of a gradual ventilation outward from the body.

The cloths more directly suitable for frocks and overalls are those of the lighter weights, self-coloured in agreeable tones of green, brown, blue, and red. There is a choice of "art colours," and there are mixture shades of blue and drab grey and of black and white. As between colourings the choice is one of taste rather than economy; but when the choice is unfettered discretion may be shown in war-time by preferring those colourings which can best be recommended on the score of durability. Nobody is immune from the shortage of dyestuffs, and there are always shades more dependable in point of fastness than others. In general, the more neutral the colour the less is any loss of tone likely to manifest itself.

In the Ruskin cloths the yarns are well twisted, and the structures adopted in weaving are the simplest of their kind. Garments made from them will in most cases be loosely fitting, and the cloths are of the sort that will regain their shape automatically should any bagging or distortion occur in wear. As the thread structure of the dress fabrics is clearly exposed from the outset, there is no question of their growing threadbare. They are double-width (54 inches), and their general price is 4s. a yard in whole-piece lengths. The price is higher than in peace-time, but the relative advantage of buying cloths of this type is greater now than under normal conditions. These pure goods have advanced in price, but articles that are ordinarily cheaper in first cost have advanced proportionately more, and some of them have also undergone changes for the worse in respect of quality.

